# Preparation of Uniform-Pore Ceramics from Highly Stable Emulsions via the Sol–Gel Method

**DOI:** 10.3390/gels11080638

**Published:** 2025-08-12

**Authors:** Alena Fedoročková, Dana Ivánová, Gabriel Sučik, Martina Kubovčíková

**Affiliations:** 1 Institute of Materials, Faculty of Materials, Metallurgy and Recycling, Technical University of Košice, Letná 9, 042 00 Košice, Slovakia; dana.ivanova@tuke.sk (D.I.); gabriel.sucik@tuke.sk (G.S.); 2 Institute of Experimental Physics, Slovak Academy of Sciences, Watsonova 47, 040 01 Košice, Slovakia; kubovcikova@saske.sk

**Keywords:** soft template, microemulsion, high kinetic stability, sol–gel method, high-purity alumina, uniform pore size

## Abstract

A facile and cost-effective sol–gel method for the synthesis of uniformly porous alumina (Al_2_O_3_) was developed using stable CTAB/hexanol/water microemulsions as soft templates. The phase behavior of the ternary system was investigated to identify compositions that form kinetically stable microemulsions, with an optimal ratio of 7.5 wt.% CTAB, 5 wt.% hexanol, and 87.5 wt.% water exhibiting minimal droplet size variation over one week. Gelation was induced by partial neutralization to pH 4.2 with ammonium carbonate, promoting the formation of polynuclear Al species and enabling the uniform entrapment of hexanol droplets. Lyophilization preserved the porous network, and calcination at 500 °C yielded η-Al_2_O_3_ with a large specific surface area (~225 m^2^·g^−1^) and a narrow mesopore size distribution centered around 100 nm, consistent with the original droplet size. Mercury porosimetry and SEM analyses confirmed a highly porous, low-density material (0.75 g·cm^−3^) with an interconnected pore morphology. This scalable synthesis method, supported by the high kinetic stability of the microemulsion, provides sufficient processing time and eliminates the need for post-synthesis purification. It shows strong potential for producing advanced alumina materials for use in energy storage, catalysis, and sensor applications.

## 1. Introduction

Porous ceramic materials have attracted considerable attention as promising candidates for addressing some of the most pressing challenges of the twenty-first century. Their applications span a wide range of fields, including CO_2_ sequestration, hydrogen storage, catalysis and separation, solar energy conversion, therapeutics (e.g., drug delivery), water purification, and more [1,2,3]. To meet the specific demands of these applications, porous ceramics must exhibit carefully tailored structural, textural, and morphological features to ensure the optimal performance [2,4,5,6,7]. Consequently, the development of synthetic strategies that enable precise control over porosity, pore morphology, and size distribution is of great industrial significance.

Among the most widely used approaches to achieve such control are replica methods, sacrificial templating, and direct foaming techniques [8,9]. These methods offer flexibility in tuning pore characteristics—such as size, shape, and spatial distribution—and can be applied to ceramics with diverse chemical compositions. Nevertheless, despite their broad applicability, these techniques often fall short of providing the level of control over porosity and morphology required to meet stringent industrial standards. An intriguing alternative that offers improved control over the resulting porous structure is the sacrificial template technique, which employs volatile oils as the sacrificial (pore-forming) phase [6,8,9,10,11]. The emulsion-templating method involves preparing an emulsion and subsequently removing the dispersed droplets through drying and heat treatment [12]. The main advantages of this approach include

(a) the ease with which the template can be incorporated into the continuous phase by simple agitation or mixing,
(b) the ability to achieve very small droplet/pore sizes when using immiscible liquids with low interfacial tension, and
(c) the mild conditions required for template removal [8].

Moreover, emulsion-mediated synthesis is regarded as a highly flexible tool for structural control, enabling the production of a wide variety of porous materials with a broad range of pore sizes [9,10]. A well-established technique for producing solids with a macroporous architecture combines the sol–gel process with emulsions used as a soft template [8,10,12,13]. This method involves emulsifying a solution of precursors, followed by gelling the sol around the oil droplets [8,13]. The addition of a surfactant to the emulsion not only reduces the droplet size but also enhances the kinetic stability of the emulsion by stabilizing the oil–water interface, thereby preventing droplet agglomeration [10]. This approach is attractive both scientifically and industrially, as it is rapid, adaptable to various chemical compositions, provides high yields, and requires relatively simple equipment [8]. In addition, it enables the precise tuning of pore sizes within a predetermined range by adjusting the emulsification conditions to produce droplets of varying sizes. The subsequent removal of the oil droplets yields ordered materials with porosities of up to 90% and pore sizes ranging from 50 nm to 10 μm [10,13].

Porous alumina is one of the most important options among functional ceramic materials, offering a unique combination of a large surface area, excellent thermal and chemical resistances, and intrinsic acid–base properties. These characteristics make it highly suitable for a wide range of advanced applications, including catalyst supports, sensors and electronic devices, biomedical uses, and energy storage and conversion [4,6,13,14,15]. Recently, several methods have been reported for the preparation of ordered macroporous alumina or alumina-based ceramics using the emulsification of ceramic suspensions with various organic phases as pore-forming templates [14,16,17]. However, producing porous materials with reproducible tolerances typically requires prolonged homogenization—up to 24 h—of the suspension by ball milling, along with the use of dispersants or deflocculants to prevent particle agglomeration [5,9,14,16,17,18]. Additionally, to prevent microstructural collapse during the subsequent drying of the gels and to ensure successful removal of the sacrificial phase—often hindered by the low level of interparticle contact within the suspension—the addition of a binder is necessary [14,17].

Emulsion-mediated synthesis of porous alumina with uniform pores utilizes emulsions—typically oil-in-water (O/W) systems—as templates to generate well-defined porous structures in aluminum oxide (Al_2_O_3_). However, the synthesis of alumina with a uniform micro/mesoporous structure typically proceeds via the condensation of hydrolyzed alkoxide precursors, which requires strict control over the reaction chemistry due to challenges associated with using an aqueous phase [9,10,12,15,19,20,21,22]. Alkoxide precursors are highly reactive with water [10,20] and must be pretreated before forming the oil-in-water (O/W) emulsion. Even with such precautions, premature gelation of the emulsion may occur before the completion of the injection (pore-forming) process [19]. To mitigate the reactivity of metal alkoxides, lower-molecular-weight alcohols [10,20] are typically used as solvents, and hydrolysis to aluminum hydroxide is achieved through the controlled addition of water [20,21]. However, large quantities of alcohol can destabilize the emulsion, as alcohol tends to promote mixing between oil and water [21]. Consequently, forming stable emulsions from alkoxide solutions presents two main challenges: first, identifying an emulsion system in which water is replaced by another polar solvent (e.g., formamide) [23] and second, developing a sol–gel process that can proceed in this polar liquid instead of alcohol [9,21]. Entrapment of the dispersed oil droplets by the continuous phase occurs during the sol–gel transition of the system, which can be induced, for example, by a rise in pH [20]. In the sol–gel-mediated synthesis of macroporous ceramics from aqueous alumina suspensions, various mucopolysaccharides are added as gelling agents to the O/W alumina slurry emulsions to initiate gelation [6,9,18].

Despite the wide range of published procedures for fabricating porous alumina via emulsion templating, these methods still present significant limitations [12]. They are often complex, requiring not only emulsifying and sacrificial phases but also incurring high equipment and energy costs associated with suspension homogenization (e.g., ball milling) [5,9,14,16,18]. The processes are costly—not only due to the use of expensive precursors and solvents, such as alkoxides, ethanol, and formamide [6,10,15,19,20,22,23,24]—but also because of the need for various additives, including dispersants, binders, and gelling or complexing agents [5,6,9,14,16,17,18,20], some of which may adversely affect the final product [12]. Moreover, although these procedures are often described as simple, efficient, cost-effective, and environmentally friendly, they tend to be time consuming. For example, preparing a homogeneous sol may require up to 5 h of stirring [7]; slurry homogenization can take up to 24 h [5,9,14,16,18], and the removal of the oil phase by Soxhlet extraction may take up to 8 h [9]. Additional limitations include the low kinetic stability of emulsions (less than 3 min) [10], as well as the use of hazardous substances, such as carcinogens (e.g., naphthalene) [18,24] and neurotoxins (e.g., toluene) [9,25,26], which further hinder the practical applicability of these approaches. Besides that, the synthesis of ordered porous Al_2_O_3_ typically involves solutions with very low concentrations of Al^3+^ salts [27], resulting in low yields—a significant challenge for large-scale production, despite the favorable properties of the final material.

In light of current requirements, there is increasing emphasis on fast, robust, affordable, reproducible, and straightforward procedures that avoid costly operations and the use of highly reactive or toxic substances. In this paper, we present a simple sol–gel emulsion-mediated synthesis of mesoporous alumina from a concentrated aqueous aluminum nitrate solution, using hexanol and cetyltrimethylammonium bromide (CTAB) as pore-structure-directing agents. The tunable composition of the selected system is expected to produce droplets or micelles of varying sizes, thereby enabling the formation of mesoporous materials with tailored pore sizes for specific applications. Importantly, the kinetic stability of the prepared emulsions was identified as a key factor, ensuring sufficient handling time for practical implementation and potential large-scale production.

## 2. Results and Discussion

### 2.1. Preliminary Study of Emulsion Formation and Kinetic Stability

The study of phase behavior is a critical step in the characterization of emulsions. To this end, a series of preliminary tests was conducted to identify the stable region of CTAB/hexanol/water emulsions at different weight ratios (as specified in Table 1), with the goal of determining the most suitable composition for the synthesis of ordered porous alumina.

The phase boundaries between the single-phase and multiphase regions were determined macroscopically by observing the transition from transparent to turbid dispersions, which gradually underwent phase separation during long-term storage, as visible to the naked eye.

**Figure 1:**
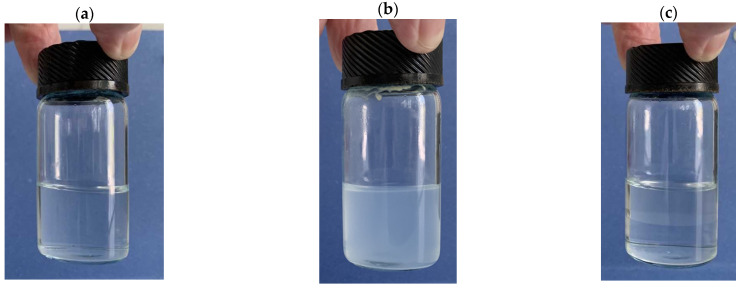
Images of samples prepared with various weight ratios of CTAB/hexanol/water, showing (**a**) microemulsion, (**b**) emulsion, and (**c**) phase separation.

**Figure 2:**
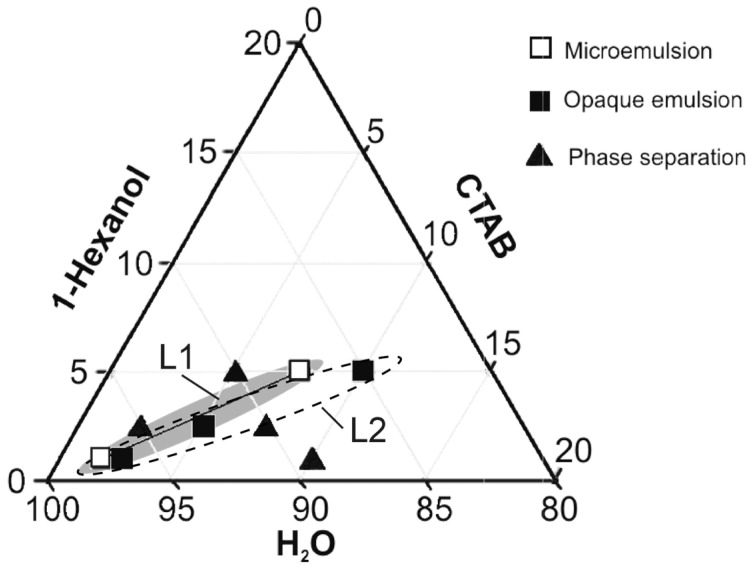
Ternary phase diagram of the studied CTAB/hexanol/water system.

The formation of a stable microemulsion, characterized by the system’s typical transparency (Figure 1a), is indicated by empty squares in the ternary phase diagram shown in Figure 2. Compositions forming opaque emulsions (Figure 1b) are marked with filled squares, while those that underwent phase separation (Figure 1c) are marked with filled triangles in Figure 2.

The arrangement of individual points in the phase diagram (Figure 2) reveals that microemulsions form within the L1 region, where the mass ratio of CTAB to hexanol ranges from 1.5 to 1.75 (as shown in Table 1). As the CTAB-to-hexanol ratio increases beyond this range (1.75 < CTAB/hexanol < 2.5), the transparent microemulsion region (L1) transitions to a broader emulsification zone (L2). This zone is characterized by larger, irregularly distributed, interconnected domains resulting from the elongation of isolated hexanol droplets [10]. This morphological change is visually reflected by the slightly opalescent appearance of the samples. Outside this optimal range—specifically, at near-equivalent ratios (CTAB/hexanol < 1.1) or when CTAB is present in significant excess (more than 2.5 times)—phase separation occurs.

### 2.2. Thermodynamic Size Distribution of Droplets Plotted as a Function of Time

**Figure 3:**
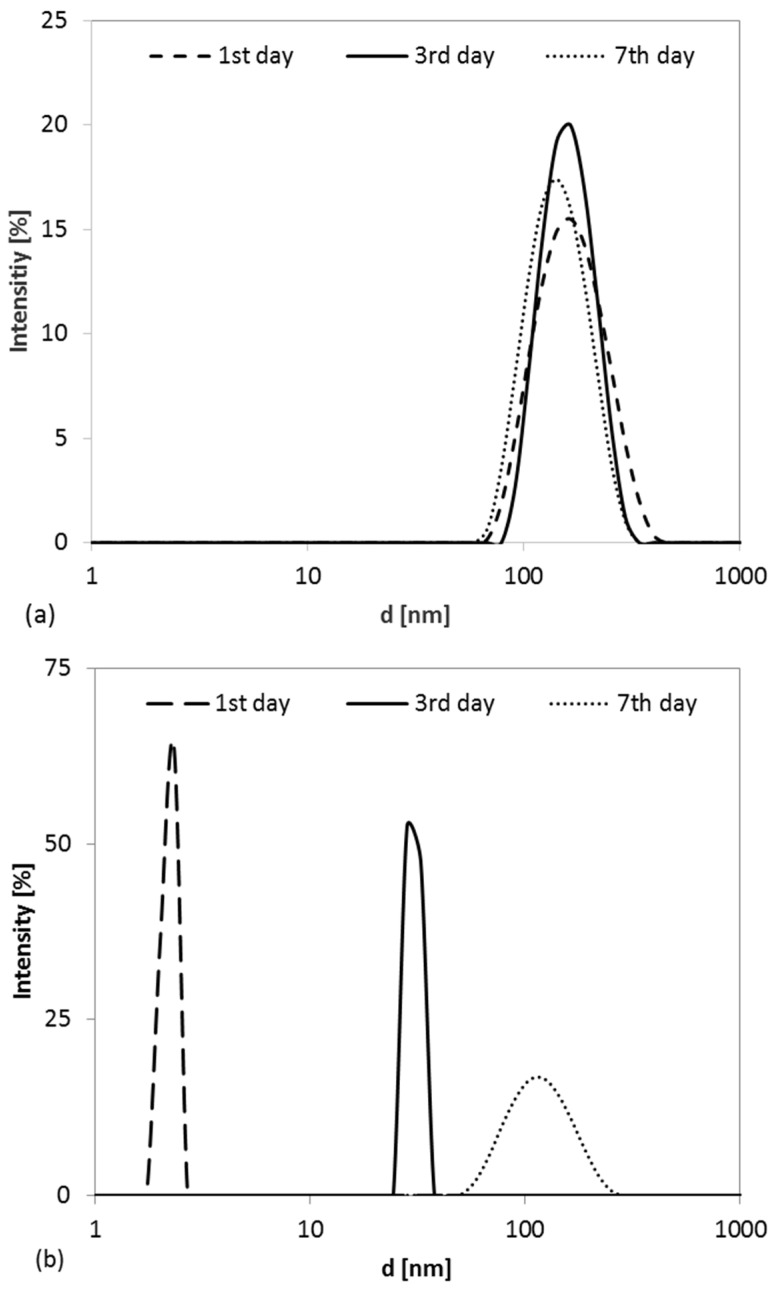
Evolution of the thermodynamic size distribution of hexanol droplets over time for microemulsions composed of (**a**) 7.5 wt.% CTAB, 5 wt.% hexanol, and 87.5 wt.% H_2_O and (**b**) 1.75 wt.% CTAB, 1 wt.% hexanol, and 97.25 wt.% H_2_O.

The systems that did not exhibit phase separation (L1 and L2, Table 1) were monitored microscopically using dynamic light scattering (DLS) over a period of seven days. Based on the DLS evaluation, the kinetically unstable emulsion in region L2—characterized by a polydisperse droplet size distribution—was excluded from further study. In contrast, the monodisperse systems in the L1 region were investigated in greater detail, specifically the compositions of 7.5% CTAB, 5% hexanol, and 87.5% H_2_O and 1.75% CTAB, 1% hexanol, and 97.25% H_2_O. Repeated DLS measurements performed on days 1, 3, and 7 after preparation are shown in Figure 3. The temporal evolution of the hydrodynamic diameter of the hexanol droplets (Figure 3a) for the 7.5% CTAB, 5% hexanol, and 87.5% H_2_O composition confirmed the formation of a kinetically stable, monomodal emulsion. The primary peak hydrodynamic diameters (by intensity) were 173.1 nm on day 1, 164.1 nm on day 3, and 148.7 nm on day 7. These results are consistent with preliminary findings and confirm the stability of the system over one week. In contrast, the system with 1.75% CTAB, 1% hexanol, and 97.25% H_2_O (Figure 3b), characterized by a higher CTAB/hexanol weight ratio (1.75), exhibited progressive growth in the droplet size over time—from 2.3 nm on day 1 to 28.2 nm on day 3, and reaching 105.7 nm by day 7. This temporal instability is attributed to the gradual coalescence of small droplets into larger ones, as evidenced by the broadening of the size distribution. In the phase diagram (Figure 2), this behavior suggests a transition from a microemulsion (L1) to a conventional emulsion (L2).

According to these results (Figure 3), the composition with 7.5% CTAB, 5% hexanol, and 87.5% H_2_O exhibited sufficient kinetic stability, maintaining a consistent droplet size distribution for at least one week.

To quantitatively assess the kinetic stability of the 7.5% CTAB, 5% hexanol, and 87.5% H_2_O system, the coalescence rate coefficient (k_coal_) was calculated based on the time-dependent decrease in the droplet density, as derived from DLS data. The kinetics of coalescence were evaluated using the Smoluchowski model, assuming binary coalescence in a dilute system. Additionally, changes in the polydispersity index (PDI) were monitored over the same period to detect any broadening of the size distribution, which could indicate destabilization mechanisms, such as coalescence or Ostwald ripening. Table 2 presents a summary of the DLS-derived parameters and relative coalescence values used to evaluate the emulsion stability over time.

The Z-average values, ranging from 119 to 140 nm, confirm the nanoscale nature of the droplets. The polydispersity index (PDI) indicates that the sample measured on day 3 (PDI = 0.177) exhibited the narrowest and most uniform droplet size distribution. In comparison, samples measured on day 1 and day 7 (PDI = 0.222 and 0.236, respectively) showed slightly broader distributions, reflecting moderate polydispersity. The enhanced uniformity observed on day 3 may contribute to improved system stability and increased suitability for applications requiring precise control over pore dimensions.

Furthermore, the negative value of k_coal_ (~−1.15 × 10^−6^ arbitrary units) and the minimal change in the PDI over seven days (from 0.222 to 0.236) confirm the high kinetic stability of the formulation, with negligible coalescence under the tested storage conditions. These findings validate the selected composition as an effective precursor for the synthesis of Al_2_O_3_ with a uniform pore size distribution. From a processing standpoint, this level of temporal stability offers a sufficiently long working window for practical or industrial applications.

### 2.3. Porous Characteristics of Alumina Prepared from Stable Emulsions via the Sol–Gel Technique

In aqueous media, Al^3+^ ions exhibit a strong tendency to hydrolyze (Equation (1)) [28] as follows:

(1) $${pAl}^{3+}\left(aq\right)+qH_{2}O\left(l\right)={{Al}_{p}{\left(OH\right)}_{q}}^{\left(3p-q\right)+}\left(aq\right)+qH^{+}\left(aq\right).$$

This hydrolysis leads to the formation of mononuclear hydroxo species (for p = 1 and q = 1–4; ${{Al(OH)}_{1}}^{2+}{{,\;Al(OH)}_{2}}^{+},\;{{Al\left(OH\right)}_{3}}^{0}(s),{{\;\mathrm{and}\;Al(OH)}_{4}}^{-}$) generated during the initial stages of the reaction. These species readily condense to more stable polynuclear complexes—such as ${{Al}_{2}{\left(OH\right)}_{2}}^{4+}$, ${{Al}_{3}{\left(OH\right)}_{4}}^{5+}$, and ${{Al}_{13}{\left(OH\right)}_{32}}^{7+}$—in which the aluminum centers are interconnected by hydroxo bridges [28]. Studies investigating the effect of pH on the distribution of Al^3+^ hydrolytic species at various ionic strengths have shown that ${{Al}_{3}{\left(OH\right)}_{4}}^{5+}$ reaches its maximum concentration at approximately pH 4, while thermodynamic models suggest that ${{Al}_{13}{\left(OH\right)}_{32}}^{7+}\;$ becomes the predominant species in the pH range 4–5 [28]. In our study, the partial neutralization of the sol to pH 4.2, using (NH_4_)_2_CO_3_, was designed to favor conditions under which the formation of Al_13_ species is expected according to these literature-based models. Although the presence of ${{Al}_{13}{\left(OH\right)}_{32}}^{7+}$ under our specific experimental conditions was not directly confirmed by techniques such as NMR, its proposed predominance serves as a plausible explanation for the enhanced gelation behavior observed. The formation of this particularly stable polynuclear species is believed to facilitate the development of a three-dimensional Al_2_O_3_ precursor network, effectively entrapping hexanol droplets within the evolving gel structure.

To preserve the porous structure—and ensure that pore sizes correspond to the original droplet dimensions—the liquid components were removed via lyophilization, thereby avoiding structural collapse during drying [8,9]. An additional advantage of using (NH_4_)_2_CO_3_ for pH adjustment is that upon calcination of the dried gel, the base decomposes completely. This eliminates the need for a post-synthesis leaching step to remove residual salts, simplifying the overall process and substantially reducing solvent waste.

The suitable calcination temperature for preparing active alumina with a nanoporous structure was determined using TG-DTA analysis.

**Figure 4:**
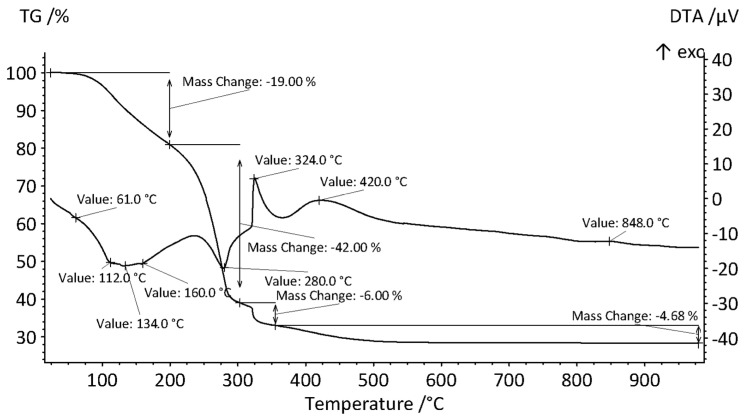
TG-DTA curves of the prepared alumina gel precursor.

Simultaneous thermal analysis (STA) of the dried gel shown in Figure 4—combining differential thermal analysis (DTA) and thermogravimetric analysis (TGA)—reveals several thermal events, as evidenced by mass loss and/or thermal effects (exothermic or endothermic). In multicomponent systems, interactions between individual components can influence the thermal behavior, leading to deviations from the characteristic temperatures reported for processes such as dehydroxylation, melting, decomposition, and crystallization.

During linear heating of the dried gel, the following events are observed:

- A triplet of small, overlapping endothermic peaks at 112 °C, 134 °C, and 160 °C corresponds to the desorption of water, gel dehydration, phase transitions, and the melting of the secondarily formed product (NH_4_NO_3_). These peaks appear superimposed on a broad endothermic background that begins at approximately 61 °C, attributed to the evaporation of residual hexanol. This process is accompanied by a mass loss of approximately 19%;
- A pronounced endothermic peak at 280 °C, beginning at around 250 °C, corresponds to the melting and simultaneous thermal decomposition of CTAB and NH_4_NO_3_, accompanied by a mass loss of approximately 42% [29,30];
- The gaseous decomposition products of CTAB and NH_4_NO_3_ react violently (explosively) in the presence of atmospheric oxygen, producing a sharp exothermic peak at 324 °C. The TG curve indicates a weight loss of 6%, which is attributed to the combustion of carbon soot. The gaseous reaction products include water vapor, CO_2_, N_2_, and Br_2_;
- The rigid residue consists of partially hydrated Al_2_O_3_ in the form of AlOOH, as evidenced by a broad exothermic peak beginning at approximately 374 °C. This peak corresponds to the phase transition to η-Al_2_O_3_ [31] and is accompanied by a weight loss of about 5%;
- The transformation of η-Al_2_O_3_ to θ-Al_2_O_3_ is marked by a small exothermic peak with a maximum near 850 °C.

These findings support using 500 °C as the initial calcination temperature for producing nanoporous alumina.

**Figure 5:**
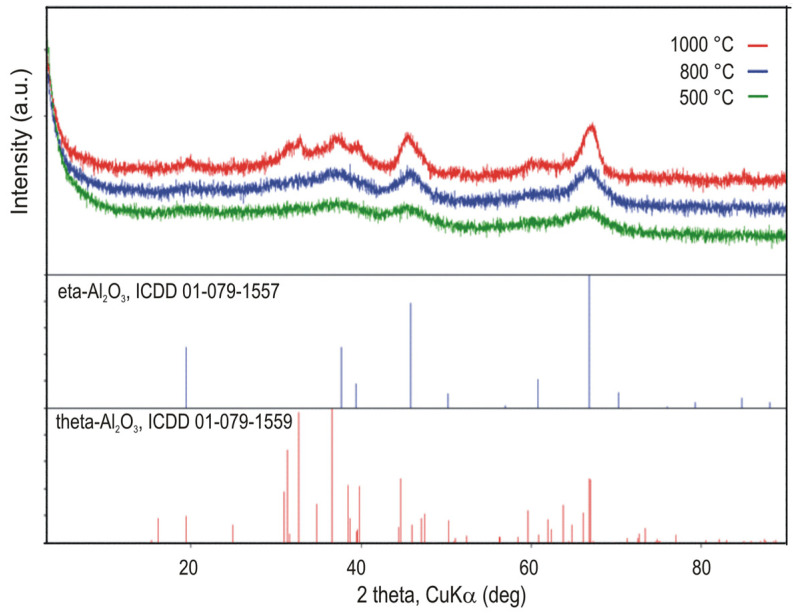
XRD patterns of the alumina samples calcined at various temperatures.

The phase identification of the calcined samples at 500, 800, and 1000 °C was carried out-using X-ray diffraction (XRD), with diffraction patterns recorded at room temperature, as shown in Figure 5.

X-ray diffraction (XRD) analysis showed that samples of dried gel calcined at 500 °C and 800 °C exhibited a quasi-amorphous structure, displaying broad, weak reflections at 2θ angles of 19.41° (1,1,1), 37.67° (3,1,1), 45.83° (4,0,0), and 66.82° (4,4,0), corresponding to the η-Al_2_O_3_ phase (ICDD #01-079-1557), space group Fd-3m [32,33]. Upon sintering at 1000 °C, partial transformation to the θ-Al_2_O_3_ phase (space group C2/m) was indicated by the emergence of sharper peaks at 19.54° (−2, 0,1), 31.46° (−4, 0,1), 32.78° (0,0,2), 36.73° (1,1,1), 38.67° (4,0,1), 44.84° (−1, 1,2), 59.90° (−3, 1,3), and 67.31° (5,1,2) (ICDD #01-079-1559). Both η- and θ-Al_2_O_3_ phases coexisted after sintering, as evidenced by asymmetric peak shapes at 2θ angles of 44.84° and 67.31°, indicating overlapping contributions from both phases. For reference, standardized diffraction patterns for the phase identification of η-Al_2_O_3_ and θ-Al_2_O_3_ are shown at the bottom of Figure 5.

The pore architectures of materials such as metal oxides, catalysts, and ceramics often undergo substantial transformation upon calcination, particularly at elevated temperatures. In templated systems, high-temperature treatment can lead to the collapse of fragile pore frameworks, resulting in the formation of larger, less uniform macropores. This structural degradation typically reduces the total porosity and surface area, thereby diminishing the material’s reactivity and overall performance [34]. Calcination at around 500 °C is widely regarded as an optimal condition for preserving well-defined mesoporous structures with narrow pore size distributions across a range of materials. At this temperature, organic templates can be efficiently removed without causing significant pore collapse or compromising the integrity of the mesostructure [35]. Therefore, calcination at 500 °C was selected as the most appropriate condition to retain the ordered porous architecture and ensure a uniform pore size distribution in the synthesized alumina.

**Figure 6:**
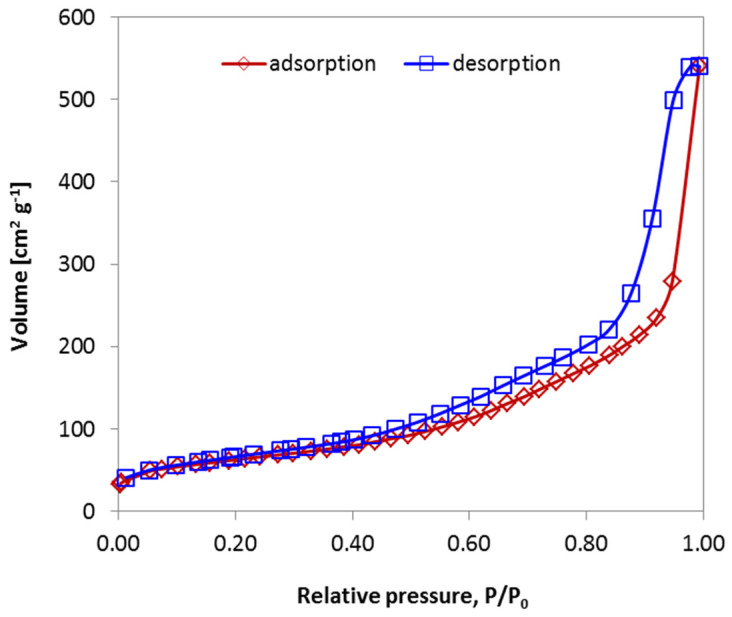
Nitrogen (N_2_) adsorption–desorption isotherms of alumina calcined at 500 °C.

The textural properties of the alumina calcined at 500 °C were evaluated immediately after thermal treatment, using nitrogen (N_2_) adsorption–desorption measurements, as shown in Figure 6.

**Figure 7:**
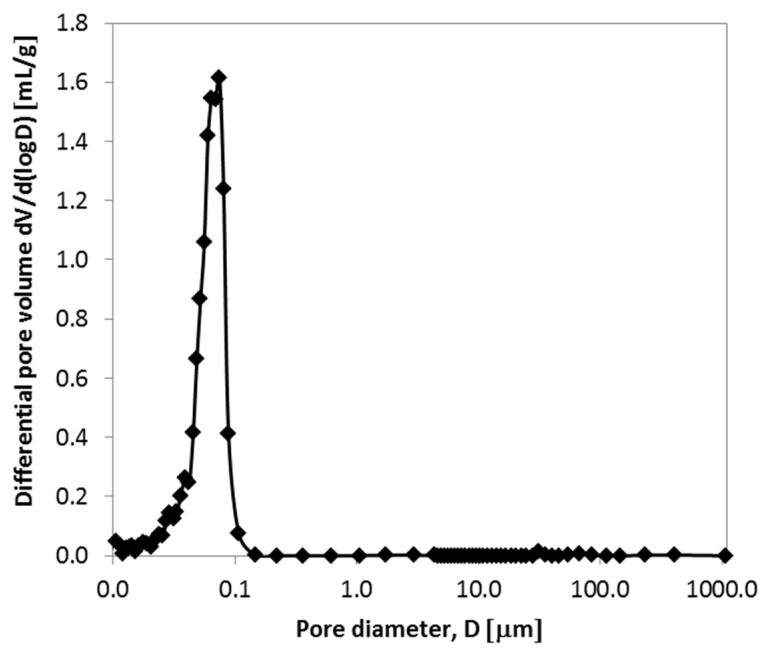
Pore size distribution obtained from high-pressure mercury intrusion porosimetry analysis of alumina calcined at 500 °C.

The shape and position of the isotherm shown in Figure 6, characterized by a distinct hysteresis loop closing at a relative pressure of approximately 0.4, confirm the presence of mesopores. Analysis of the adsorption branch, using the linearized BET equation within the standard relative pressure range (0.05 ≤ p/p_0_ ≤ 0.35), yielded a specific surface area of 225 ± 3 m^2^·g^−1^. This large surface area is primarily attributed to the voids generated during the oxidative degradation of 1-hexanol and CTAB, the decomposition of NH_4_NO_3_, and the dehydroxylation of boehmite to η-Al_2_O_3_. The external surface area (S_ext_), calculated from the mesopore volume, was found to be 217 m^2^·g^−1^. The close correspondence between the BET surface area (S_BET_) and S_ext_ indicates that the majority of the surface area is associated with the external surfaces of the porous alumina particles rather than with microporous contributions—highlighting the presence of a well-developed mesoporous structure [36]. The mesopore size distribution was evaluated using high-pressure mercury intrusion porosimetry, with the results presented in Figure 7. The pore size distribution (Figure 7), obtained from the derivative of the volume–pressure curve, demonstrates a uniform microstructure, featuring a narrow, monomodal distribution centered at around 100 nm. This finding aligns well with the hydrodynamic average size of the hexanol droplets (~160 nm) observed in Figure 3a. The slight reduction in the pore size relative to the original droplet size is attributed to shrinkage during thermal treatment. This strong correlation confirms the suitability of the chosen emulsion composition for producing uniformly porous alumina materials. The material exhibits a remarkably low apparent density of 0.75 g·cm^−3^ and an open, interconnected porosity of approximately 78%, as determined from three independent mercury intrusion porosimetry (Hg porosimetry) measurements. These characteristics indicate the formation of an ultralight, highly porous Al_2_O_3_ ceramic, making it a promising candidate for advanced applications across various industrial sectors—particularly in energy storage. Moreover, its low permeability makes it suitable for use as a barrier material in hydrogen storage systems or as a support in catalytic processes.

**Figure 8:**
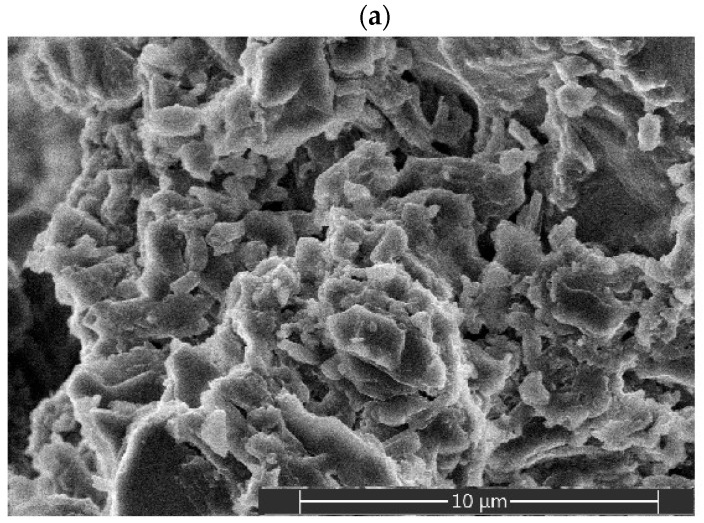

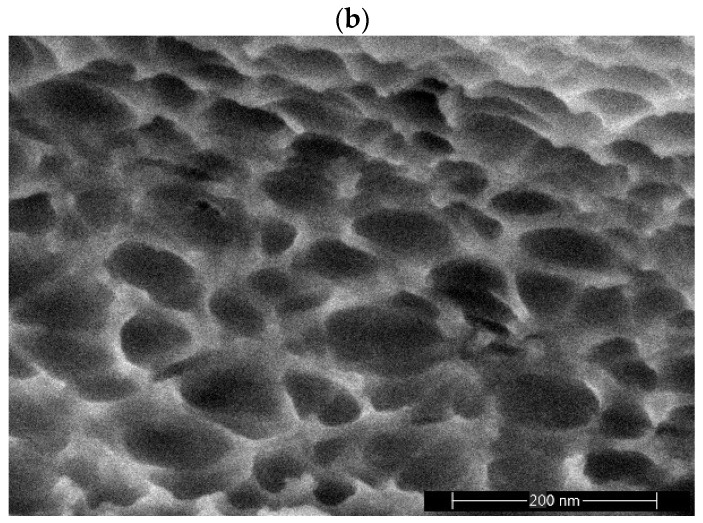
SEM micrographs of the alumina sample: (**a**) dried gel; (**b**) sample after calcination at 500 °C.

The pore size distribution observed using mercury intrusion porosimetry is further supported by the SEM images shown in Figure 8, which compare the sample morphologies before and after calcination. Figure 8a depicts the dried gel at low magnification (4000×), revealing a highly roughened, flower-like structure composed of thin plates up to 300 nm thick, separated by narrow gaps. In contrast, Figure 8b shows a high-magnification view (60,000×) of the particle surface after calcination, highlighting a uniform nanostructure of elongated spheroidal pores with diameters ranging from 60 to 110 nm. These pore dimensions closely correspond to the pore size distribution shown in Figure 7. The elongation of the pores is likely caused by anisotropic stresses during thermal treatment, which arise from the removal of volatile components, directional shrinkage, grain growth, and phase transformations [34].

Ordered mesoporous alumina materials with large surface areas and uniform pore size distributions are typically prepared through two primary strategies: nanocasting using rigid templates and self-assembly directed by surfactant aggregates. Among these, the evaporation-induced self-assembly (EISA) method stands out for its simplicity and speed in producing highly ordered structures at the nanoscale [7]. However, EISA generally relies on dilute solutions and volatile, non-aqueous solvents, such as ethanol, to control the hydrolysis and condensation rates of aluminum precursors, like aluminum isopropoxide [7,24]. These conditions can impair surfactant function and hinder effective interaction between precursor species and templating agents, limiting the efficiency of mesostructure formation [7]. Besides that, the self-assembly process is primarily activated during solvent evaporation, as increasing surfactant concentration and precursor reactivity drive the organization of mesophases. As a result, several factors—including the temperature, humidity, and evaporation rate—can significantly influence the final material architecture [7]. A comparison of the preparation conditions and properties of Al_2_O_3_ obtained using different methods, in the context of the present study, is summarized in Table 3.

In contrast to the methods described above, the procedure presented herein for the synthesis of Al_2_O_3_ with a uniform pore structure is based on concentrated aqueous solutions of inorganic aluminum salts. These salts can be replaced with refined leachates derived from secondary aluminum-based raw materials, such as industrial wastes (e.g., slurries and sludges). The use of concentrated solutions leads to higher yields and, when combined with the cost of the aluminum precursors—aluminum isopropoxide (EUR 31.2/100 g, ≥98%; Sigma-Aldrich, Saint Louis, MO, USA) versus aluminum nitrate (EUR 2.6/100 g, p.a.; Centralchem, Bratislava, Slovakia)—represents a key factor in the feasibility of large-scale production. Based on the prices of the input materials, the estimated cost of the chemicals required to synthesize 1 g of Al_2_O_3_ using the selected microemulsion composition is approximately EUR 0.50. This method also offers a time-saving advantage, with gel preparation taking up to 0.5 h, in contrast to 5 to over 24 h for organic-precursor-based approaches [22,24].

## 3. Conclusions

The synthesis of ordered mesoporous alumina materials with large surface areas and uniform pore size distributions typically involves complex methods, such as nanocasting and evaporation-induced self-assembly (EISA). While EISA offers simplicity and rapid formation of the nanoscale order, it often requires dilute solutions and volatile organic solvents that can limit surfactant efficiency and control over the mesostructure formation. These factors, along with environmental variables, like temperature and humidity, introduce challenges in reproducibility and scalability.

In contrast, the method presented herein employs concentrated aqueous solutions of inorganic aluminum salts, enabling the production of alumina with uniform mesopores sized between 60 and 110 nm, a large surface area (~225 m^2^/g), and an interconnected porous network with an ultralow density (~0.75 g/cm^3^). This approach benefits from lower precursor costs, shorter processing times, and the potential use of industrial-waste-derived raw materials, addressing key limitations of traditional organic-solvent-based methods.

However, the current procedure’s reliance on lyophilization and specific surfactant-templating agents may limit adaptability to other precursor systems or larger-scale continuous production. Future research should focus on further simplifying processing steps, exploring alternative templating agents, and evaluating the mechanical and functional stabilities of the porous alumina under operational conditions to broaden its applicability in energy storage and catalytic technologies.

## 4. Materials and Methods

### 4.1. Materials

All the chemical reagents and solvents used in this study were of ACS grade and employed without further purification. Aluminum nitrate nonahydrate and ammonium nitrate were procured from Centralchem s.r.o. Bratislava, Slovakia a specialized chemical supplier. Hexanol (98%, density = 0.813 g·cm^−3^) and hexadecyltrimethylammonium bromide (CTAB, ≥99%) were purchased from Acros Organics (Waltham, MA, USA).

### 4.2. Preparation of Uniformly Porous Al_2_O_3_ via the Sol–Gel Processing of Stable Emulsions

A precisely weighed amount of CTAB was gradually added to the corresponding volume of hexanol at the desired weight ratio, under isothermal conditions (22 °C), with constant stirring (500 rpm) to ensure homogenization. Subsequently, a precisely measured volume of a 1.4 mol·dm^−3^ Al(NO_3_)_3_·9H_2_O solution (density = 1.253 g·cm^−3^) was added to the mixture and stirred for an additional 10 min. The prepared samples, exhibiting either characteristic transparency or forming opaque emulsions, were then subjected to DLS measurements. To evaluate the kinetic stability of the microemulsions, DLS analyses were repeated on the third and seventh days after the preparation.

For the gel preparation, a composition of 7.5 wt.% CTAB, 5 wt.% hexanol, and 87.5 wt.% H_2_O—which demonstrated a stable hexanol droplet size distribution over 7 days—was selected. The initial emulsion (pH 0.9) was adjusted by the gradual addition of a 1 mol·dm^−3^ (NH_4_)_2_CO_3_ solution at a rate of 0.5 mL min^−1^ under continuous stirring (500 rpm), until the pH reached 4.2. After standing for 30 min to promote gelation, the mixture was gently dried using lyophilization to preserve the pores. The resulting dehydrated gel was then calcined at 500 °C for 2 h at a heating rate of 3 °C·min^−1^. The calcined sample was subsequently subjected to physicochemical characterization. To identify the phase transformation, the samples were calcined at 800 °C and 1000 °C for 2 h.

### 4.3. Characterization

The hydrodynamic size of the droplets was measured using dynamic light scattering (DLS). Hydrodynamic diameters were calculated using the cross-correlation method on a Nanophox system (Sympatec GmbH, Clausthal-Zellerfeld, Germany). Size distribution measurements were performed with a Zetasizer Nano ZS instrument (Malvern Instruments Ltd., Malvern, UK) equipped with Zetasizer Software v7.13 and using cuvettes (DTS 0012). The liquid phase was removed from the gel structure by lyophilization using a vacuum freeze dryer (Model LGJ-10, Version 55C, Vekuma Machinery Co., Ltd.).

Differential and gravimetric thermal analyses (DTA–TG) were conducted using a NETZSCH STA 449 F Jupiter thermal analyzer (NETZSCH-Gerätebau GmbH, Selb, Germany) in a synthetic air (20 mL·min^−1^) and a nitrogen protective gas (20 mL·min^−1^) atmosphere. Measurements were carried out in Al_2_O_3_ (alumina) crucibles under an initial vacuum from 20 °C to 1000 °C at a heating rate of 10 °C·min^−1^. The phase structure of the calcined samples was investigated at room temperature, using a Rigaku Miniflex 600 X-ray powder diffractometer (Rigaku Corporation, Tokyo, Japan) with CuKα radiation (λ = 1.541862 Å), a Ni filter, and a scintillation detector (SC-70), starting in the range of 2θ from 3° to 90° and at step size of 0.02°; these were the scanning conditions. X-ray diffraction (XRD) data were processed and analyzed using PDXL2 software (version 2.8.4.0; Rigaku Corporation, Tokyo, Japan) in conjunction with the ICDD PDF-2 database. Textural properties were analyzed using nitrogen adsorption–desorption isotherms and a NOVA 1000e surface area analyzer (Quantachrome Instruments, Boynton Beach, FL, USA). The specific surface area was calculated via the multipoint Brunauer–Emmett–Teller (BET) method. The pore size distribution was determined using high-pressure mercury porosimetry and a PoreMaster 33 instrument (Quantachrome Instruments Ltd., USA). The powder morphology and microstructure were examined using a Quattro S environmental scanning electron microscope (Thermo Fisher Scientific, Waltham, MA, USA) operated at an accelerating voltage of 5.00 kV and a working distance of 8 mm.

## Figures and Tables

**Table 1:** Composition of the investigated ternary systems.

Emulsion Composition (wt.%)				
CTAB	Hexanol	Water	$\frac{w_{CTAB}}{w_{Hexanol}}$	Region
2.5	2.5	95	1	Phase separation
5	5	90	1	Phase separation
4	3.5	92.5	1.14	Phase separation
7.5	5	87.5	1.5	L1
1.75	1	97.25	1.75	L1
5	2.5	92.5	2	L2
10	5	85	2	L2
2.5	1	96.5	2.5	L2
7.5	2.5	90	3	Phase separation
10	1	89	10	Phase separation

**Table 2:** DLS-derived parameters and relative coalescence over time for emulsion stability evaluation.

Day	Peak Mean (nm)	Z-Avg. (nm)	PDI	Relative k_coal_ (Days 1–7) (Arb. Units)
1	173.1	139.3	0.222	-
3	164.1	140.3	0.177	-
7	148.7	119.0	0.236	~−1.15 × 10^−6^

**Table 3:** Comparison of physicochemical properties of Al_2_O_3_ prepared via different methods.

Method	Al^3+^ Source	$c_{{Al}^{3+}}$ $\left(mol\;{dm}^{-3}\right)$	Additive(s)	Treatment			Properties of Al_2_O_3_		Ref.
				Aging T (°C)/ Time (h)	Drying T (°C)/ Time (d)	Calcination T (°C)/ Time (h)	S_A_ (m^2^·g^−1^)	Pore Diameter	
EISA	Al(C_3_H_7_O)_3_	0.5 (EtOH)	Pluronic P123		60/2	400/4 900/1 1000/1	419.6 220.9 146.6	3.92 nm 3.93 nm 3.94 nm	[24]
EISA solvoth.	Al(C_3_H_7_O)_3_	0.5 (EtOH)	Pluronic P123	l80/24 100/24 130/24	60/2	400/4 900/1 1000/1	742.9 422.8 182.6	10.08 nm 5.81 nm 6.82 nm	
Sol–gel	Al(C_3_H_7_O)_3_	1.5 (H_2_O)	Pluronic P123 n-PentOH Decalin NH_4_OH	-/168	50/2	600/2	225–473	Bimodal 8–10 nm 0.1–6 μm	[13]
Sol–gel	Al(C_3_H_7_O)_3_	0.8 (H_2_O)	HNO_3_ MMA SDS APS	-/3	110	600/3	228–236	Bimodal 3.8 nm 25.7 nm	[15]
Sol–gel	Al(C_3_H_7_O)_3_	0.5 (EtOH)	Pluronic P123 PEG	-/24	60/2	450/5	199–287	13–29 nm	[20]
Sol–gel	Al(C_3_H_7_O)_3_	2 (EtOH +H_2_O)	PEO	40/24	50/2	600/2	427–675	6.5–15.1 nm	[22]
Sol–gel	AlCl_3_	- (EtOH)	PEG	40/24	50/1	600/6	273–565	4.31–14.36 nm	[6]
Sol–gel	Al(NO_3_)_3_	- (n-PrOH)	CA HMTA Carbamide EtOAc PO		80/-	500/2	110–290	0.5–120 μm	[27]
					Lyoph.		150–310	0.5–15 μm	
Sol–gel	Al(NO_3_)_3_	1.4 (H_2_O)	HexOH CTAB		Lyoph.	500/2	225	60–110 nm	This work

Abbreviations: APS, ammonium persulfate; CA, citric acid; Decalin, decahydronaphthalene; EtOAc, ethyl acetate; EtOH, ethanol; HexOH, hexanol; HMTA, Urotropin; MMA, Methyl methacrylate; PEG, polyethylene glycol; n-PentOH, n pentanol; n-PrOH, n-propanol; PEO, poly(ethylene oxide); PO, propylene oxide; SDS, sodium dodecyl sulfate; S_A_, specific surface area.

## Data Availability

This article presents the original findings of the current study. The corresponding author can provide the data discussed in this publication upon request, as it is a part of an ongoing investigation.

## Abbreviation

The following abbreviation is used in this manuscript:

CTAB	hexadecyltrimethylammonium bromide
